# Phenotypic and functional characterization of Bst^+/−^ mouse retina

**DOI:** 10.1242/dmm.018176

**Published:** 2015-08-01

**Authors:** Hamidreza Riazifar, Guoli Sun, Xinjian Wang, Alan Rupp, Shruti Vemaraju, Fred N. Ross-Cisneros, Richard A. Lang, Alfredo A. Sadun, Samer Hattar, Min-Xin Guan, Taosheng Huang

**Affiliations:** 1 Department of Pediatrics, Division of Human Genetics, University of California, Irvine, CA 92697, USA; 2 Division of Human Genetics, Cincinnati Children's Hospital Medical Center, Cincinnati, OH 45229, USA; 3 Johns Hopkins University, Department of Biology, Baltimore, MD 21218, USA; 4 Vision Science Group, Cincinnati Children's Hospital Medical Center, Cincinnati, OH 45229, USA; 5 Doheny Eye Institute, Department of Ophthalmology, University of Southern California, Los Angeles, CA 90033, USA

**Keywords:** Bst, Melanopsin, Retinal ganglion cell

## Abstract

The belly spot and tail (Bst^+/−^) mouse phenotype is caused by mutations of the ribosomal protein L24 (Rpl24). Among various phenotypes in Bst^+/−^ mice, the most interesting are its retinal abnormalities, consisting of delayed closure of choroid fissures, decreased ganglion cells and subretinal vascularization. We further characterized the Bst^+/−^ mouse and investigated the underlying molecular mechanisms to assess the feasibility of using this strain as a model for stem cell therapy of retinal degenerative diseases due to retinal ganglion cell (RGC) loss. We found that, although RGCs are significantly reduced in retinal ganglion cell layer in Bst^+/−^ mouse, melanopsin^+^ RGCs, also called ipRGCs, appear to be unchanged. Pupillary light reflex was completely absent in Bst^+/−^ mice but they had a normal circadian rhythm. In order to examine the pathological abnormalities in Bst^+/−^ mice, we performed electron microscopy in RGC and found that mitochondria morphology was deformed, having irregular borders and lacking cristae. The complex activities of the mitochondrial electron transport chain were significantly decreased. Finally, for subretinal vascularization, we also found that angiogenesis is delayed in Bst^+/−^ associated with delayed hyaloid regression. Characterization of Bst^+/−^ retina suggests that the Bst^+/−^ mouse strain could be a useful murine model. It might be used to explore further the pathogenesis and strategy of treatment of retinal degenerative diseases by employing stem cell technology.

## Abstract (precis)

**Summary:** We characterized Bst^+/−^ mice and found that pupillary light reflex was completely absent, which could be used as a readout for the efficacy of stem cell therapy in this model.

## INTRODUCTION

Belly spot and tail (Bst^+/−^) mice were first reported in 1976 as a spontaneous mutant line that emerged in an inbred C57/BlkS colony (Oliver et al., 2004). The Bst mutation has a semidominant phenotype, with heterozygous (*Bst*^+/−^) inheritance producing viable, fertile offspring, and homozygosity being lethal *in utero*. Bst^+/−^ mice have variable phenotypic traits, including a kinked tail, white spots on the feet and abdomen, dental malocclusion, ocular defects, smaller-than-normal body size, abnormalities of the spine and, occasionally, polydactyly (Epstein et al., 1986; Smith et al., 2000). The genetic mutation responsible for the Bst^+/−^ phenotype was mapped to mouse chromosome 16 (Rice et al., 1995). Specifically, it is a 4-bp deletion in the riboprotein gene *Rpl24*, which disrupts the first intron branch point and causes abnormal splicing with retention of intron 1. The resultant mutant Rpl24 protein alters ribosome biogenesis, reducing cellular protein synthesis and cell proliferation rates (Oliver et al., 2004). The Bst^+/−^ mouse has a striking ocular presentation characterized by retinal colobomas, reduced retinal ganglion cells (RGCs) and axon misrouting (Rice et al., 1997; Tang et al., 1999). These ocular features appear to be due to *Rpl24* mutation effects on retinal cell differentiation, leading to optic fissure-fusion and other developmental delays (Tang et al., 1999). Abnormal axonal migration also contributes to retinal structure abnormality in the Bst^+/−^ mouse (Rice et al., 1997).

Vascular patterning is important for organ morphogenesis and function. During eye development, the embryonic hyaloid vasculature regresses, clearing the optical path (Ito and Yoshioka, 1999), while the retinal vasculature forms concurrently (Fruttiger, 2007). In the mouse, these processes occur postnatally and are regulated by light-responsive pathways (Rao et al., 2013). A reliable blood supply is crucial for high-energy-demand organ development, such as eye development, whereas vasculature abnormalities can lead to hypoxia and a low-energy state, which can affect neuronal cell differentiation and growth of neurons, especially in the developing retina. Therefore, characterization of vasculature development in the Bst^+/−^ mouse will be important for understanding the developmental mechanisms underlying the phenotype.

Melanopsin is expressed early in murine development (Tarttelin et al., 2003), and a melanopsin-dependent fetal light response pathway regulates mouse eye development by controlling retinal vascular development (Rao et al., 2013). RGC number is reduced significantly in Bst^+/−^ mice (Oliver et al., 2004). Given recent evidence indicating that melanopsin-expressing RGCs are resistant to neurodegeneration in mitochondrial optic neuropathies (La Morgia et al., 2010), it would be interesting to determine whether any RGC subtypes are preserved and, if so, how their preservation contributes to retina function in Bst^+/−^ mice. Melanopsin^+^ RGCs, also known as intrinsically photosensitive retinal ganglion cells (ipRGCs), play a key role in the pupillary light reflex (PLR) and circadian rhythm. Given the integral involvement of ipRGCs in retina function, it is plausible to suppose that they play an important role in the retinal development of the Bst^+/−^ mouse.

The purpose of the present study was to characterize retina structure and function in Bst^+/−^ mice. Protein expression of the RGC marker Brn3a (Nadal-Nicolas et al., 2009) and of the ipRGC marker melanopsin was assessed in retina sections and whole-mounts from Bst^+/−^ and wild-type (WT) mice by immunohistochemistry (IHC). We tested whether the Bst mutation has functional effects on the PLR – a readily quantifiable behavior driven by ipRGCs – and circadian rhythm. Mitochondrial ultrastructure in RGCs was examined by electron microscopy (EM), and mitochondrial functions were also tested. Additionally, hyaloid vessel regression and superficial layer retinal angiogenesis, which are two crucial concurrent processes in retina vasculature formation, were examined in Bst^+/−^ and WT mouse pups. As most of the retinal disease models for RGC loss currently in use were generated by chemical and physical insults, there is a need for genetic mouse models of retinal disease due to RGC loss that can be used for stem cell therapy and studies of the pathogenesis of RGC loss.

**TRANSLATIONAL IMPACT**

**Clinical issue**

In humans, retinal ganglion cell (RGC) loss is associated with many conditions. Among them, autosomal dominant optic atrophy is mainly caused by mutations in the optic atrophy 1 (*OPA1*) gene, which encodes a protein that regulates mitochondria morphology. Individuals with this condition often present with central and color vision loss. Leber's hereditary optic neuropathy is the most common maternally inherited form of blindness. In addition, glaucoma is the most common RGC-loss condition, affecting over four million Americans. For all these conditions associated with RGC loss, stem cell therapy to replace damaged cells provides a promising prospective, if not the only treatment option. Animal models are important to test the efficacy of this therapy. Currently, most murine models are generated by chemical or physical methods, which have limited applicability for testing stem cell therapies. Thus, genetic murine models of retinal disease associated with RGC loss are urgently required to study retinal degeneration pathogenesis further and to investigate the potential use of stem cell therapy to treat this disease.

**Results**

This study characterized the retina of the belly spot and tail (Bst^+/–^) mouse, a spontaneous mutant line that shows several ocular defects, including reduced RGCs. In Bst^+/–^ mice, RGCs are significantly reduced in the RGC layer but melanopsin^+^ intrinsically photosensitive RGC (ipRGCs) seem preserved. Pupillary light reflex is completely absent, whereas circadian rhythm is normal. In order to further examine the pathological abnormalities of Bst^+/–^ mice, the authors performed electron microscopy in RGCs and observed abnormal mitochondria morphology with irregular borders and lacking cristae. The complex activities of mitochondrial electron transport chain were significantly decreased with different tissue specificity. Finally, the analysis of subretinal vascularization revealed delayed angiogenesis in Bst^+/–^ mice associated with delayed hyaloid regression.

**Implication and future directions**

The Bst^+/–^ mouse holds great promise for potential use as a murine genetic model of retinal degenerative disease associated with RGC loss. The defective pupillary light reflex of Bst^+/–^ mice could be used as a convenient readout for quickly screening the effectiveness of stem cell therapies, a highly useful feature of this murine model providing a unique advantage over other systems. This method would also significantly simplify the procedure for testing potential treatments and reduce the cost of such experiments. Retinal development is a complicated and poorly understood process. Data from the current study demonstrate abnormal mitochondria morphology and altered vascular patterning in the Bst^+/–^ mouse retina, suggesting these as potential underlying molecular mechanisms of RGC damage. This mouse model thus provides unprecedented opportunities and tools to investigate further the pathogenesis of retinal degenerative disease and to identify new therapeutic targets for medical intervention.

## RESULTS

### Bst^+/−^ mouse retina has apparently normal ipRGCs

**Fig. 1.**
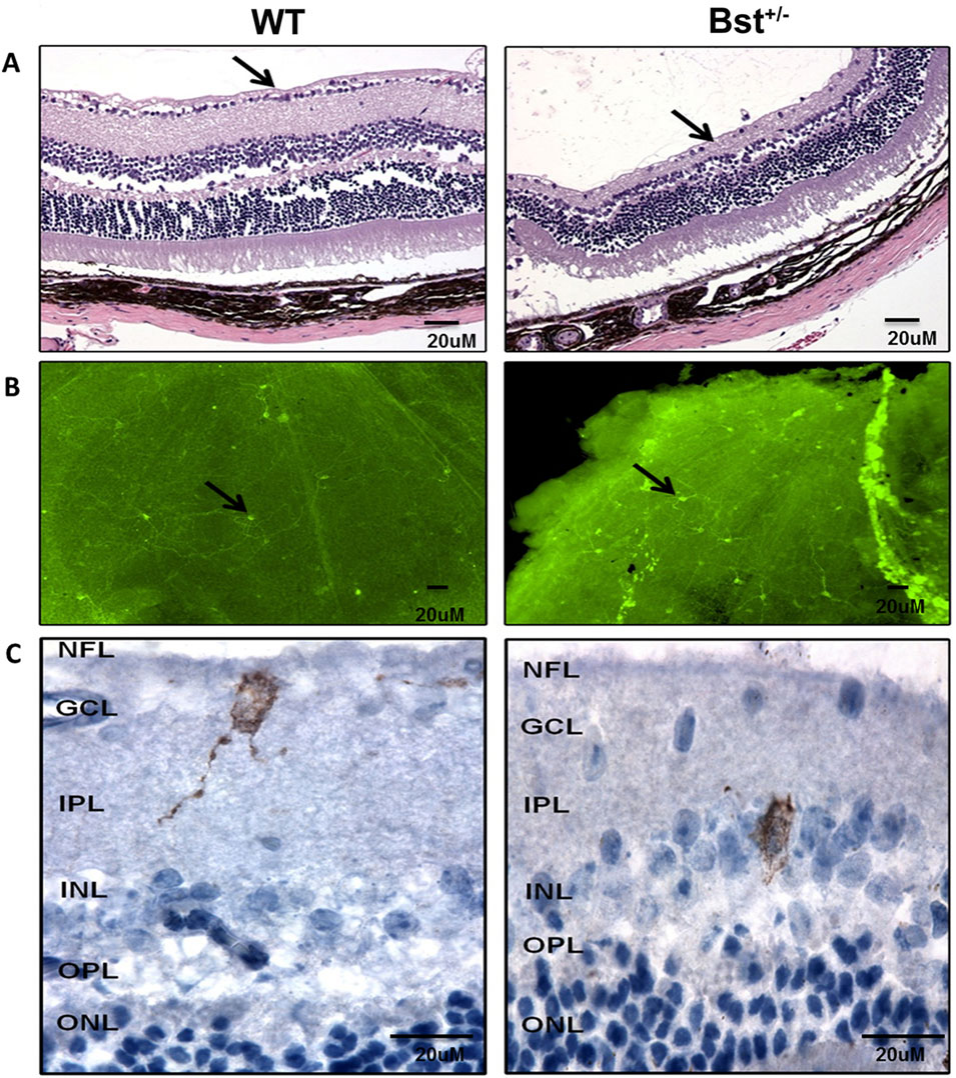
**Histological and immunohistochemical analysis of retina.** (A) Retinal sections from WT and Bst^+/−^ mice. The number of RGCs was severely reduced in Bst^+/−^ mice retinas compared with those in WT retinas (arrows, H&E-stained sections). (B) Whole-mount immunostaining of WT and Bst^+/−^ mice retinas indicated equal amount of melanopsin^+^ cells (arrows). (C) Melanopsin^+^ RGCs in Bst^+/−^ and WT mice were stained in brown by immunohistochemistry (IHC). Proceeding from the inside to the outside of the eye, clearly defined layers were labeled as: NFL, nerve fiber layer; GCL, ganglion cell layer; IPL, inner plexiform layer; INL, inner nuclear layer; OPL, outer plexiform layer; ONL, outer nuclear layer.

It has been previously reported that Bst^+/−^ mice have a significant RGCs loss. As shown in Fig. 1A, a 90% reduction in RGCs was observed in Bst^+/−^ retinas compared with WT retinas, as visualized in hematoxylin & eosin (H&E)-stained sections. The inner plexiform layer (IPL) and inner nuclear layer (INL) thicknesses were also reduced in Bst^+/−^ mice (Fig. 1A), consistent with ganglion cell loss and a decrease in the number of axons coming from the IPL and INL layers. Melanopsin^+^ cells were detectable in Bst^+/−^ retina sections (Fig. 1C). Counts of melanopsin^+^ cells in *in situ* whole-mounts of retinas from Bst^+/−^ mice were similar to those observed in WT mice (Fig. 1B).

**Fig. 2.**
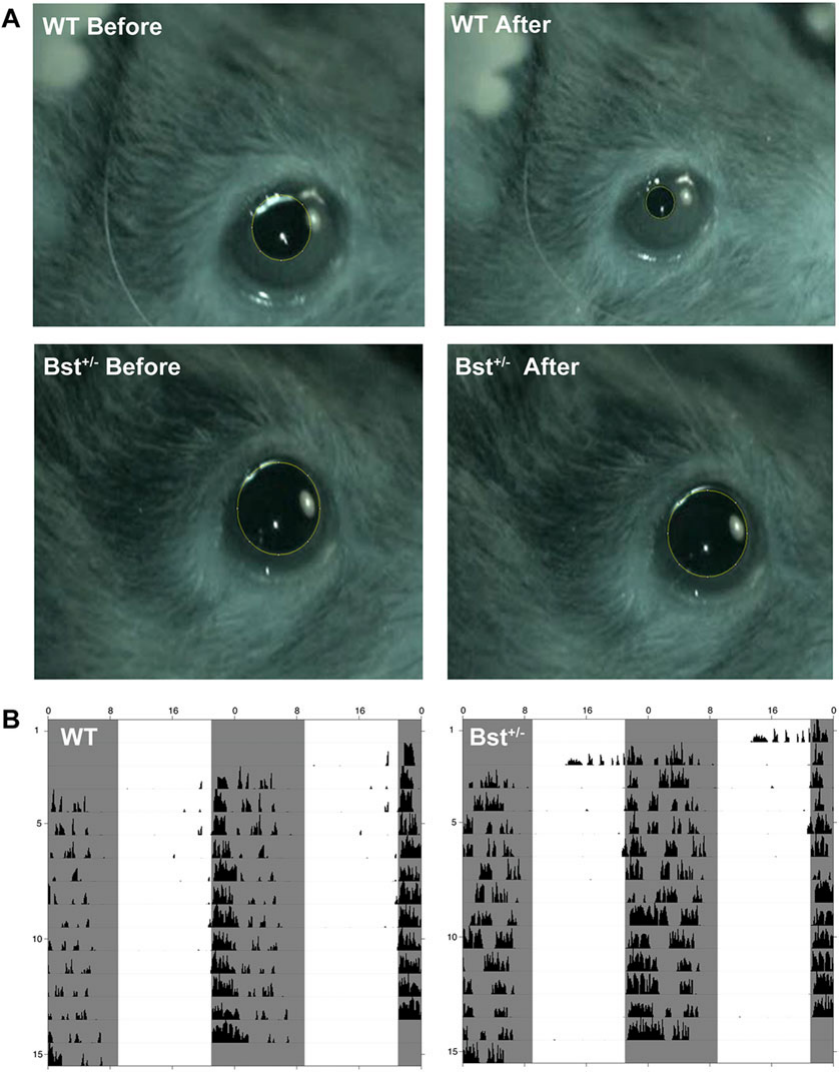
**Ocular photodetection.** (A) Exposure to a light stimulus triggered a significant decrease in pupil diameter in WT mice but not in Bst^+/−^ mice, indicating that PLR in Bst^+/−^ mice is impaired. (B) Mice actograms were used to record circadian rhythm wheel-running activity for WT and Bst^+/−^ mice. Similar wheel-running activity patterns were observed in WT and Bst^+/−^ mice, showing normal circadian rhythms preserved in Bst^+/−^ mice. The light cycles are represented by shaded areas for dark periods and unshaded areas for light periods. Activity bouts are represented as histogram bars.

To test ipRGC functions in Bst^+/−^ and WT mice, we examined their PLR and circadian rhythms. Ocular photodetection testing showed that, compared with WT mice, Bst^+/−^ mice had abnormal PLRs but similar circadian rhythms. As shown in Fig. 2A, pupils in WT mice constricted immediately upon being subjected to illumination, whereas the pupils in Bst^+/−^ mice showed very little change after illumination. Meanwhile, the wheel-running activity of Bst^+/−^ mice under light/dark schedule challenges had the same pattern as that observed for WT mice. Both WT and Bst^+/−^ mice are active in the light and inactive in the dark, suggesting that circadian rhythm physiology is preserved in the Bst^+/−^ mouse (Fig. 2B). In this experiment, the same group of mice was used in both the PLR experiment and in the subsequent circadian rhythm wheel-running activity.

### Melanopsin**^+^** RGCs are Brn3a**^–^**

**Fig. 3.**
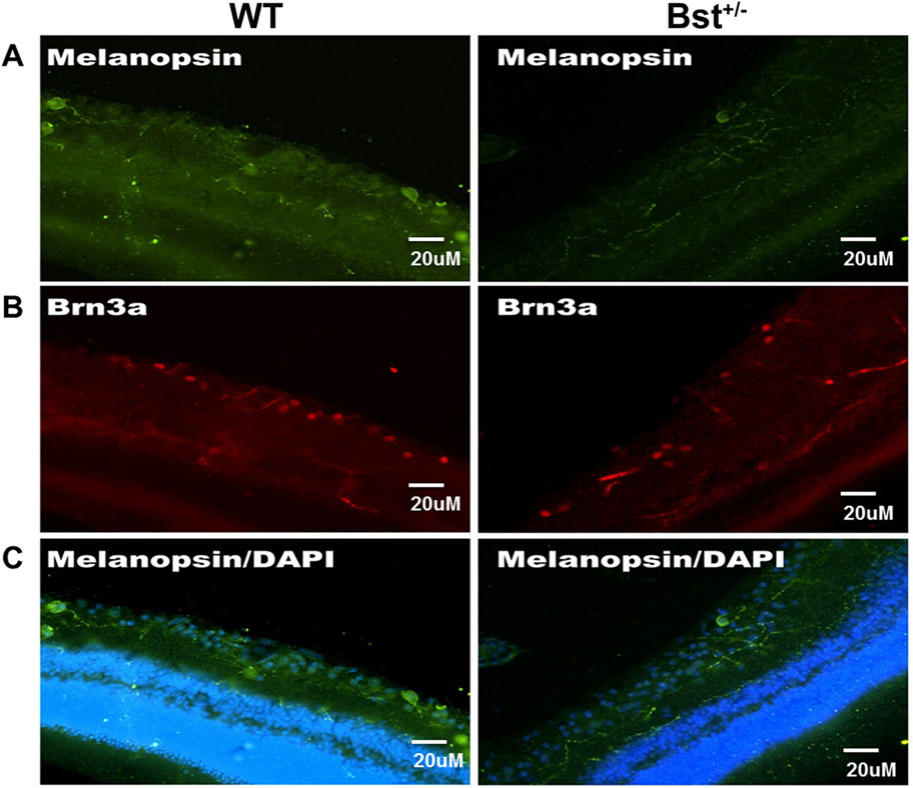
**Characterization of RGCs in WT and Bst^+/−^ retinas.** Sections of WT and Bst^+/−^ mice retinas were stained with anti-melanopsin and anti-Brn3a antibodies, and images were taken by using the same confocal microscopy parameters for primary antibody. (A) Melanopsin^+^ RGCs (arrow) in retinas harvested from Bst^+/−^ and WT mice. (B) There were fewer Brn3a^+^ RGCs in Bst^+/−^ retinas than in WT retinas. (C) Superimposed melanopsin (green) immunolabeling with DAPI nuclear counterstain (blue).

To further characterize melanopsin^+^ RGCs, sections of WT and Bst^+/−^ mouse retinas were immunolabeled for melanopsin and Brn3a. Melanopsin-immunopositive RGCs in Bst^+/−^ mouse retinas appeared very similar to those in WT mouse retinas (Fig. 3A). However, Bst^+/−^ mouse retinas have very few Brn3a^+^ cells (Fig. 3B); Brn3a-expressing RGCs innervate the retinal-hypothalmic/retinal-colicular pathway. This structural distinction is consistent with the differing appearances of the mutant versus WT retinas seen in H&E-stained sections (Fig. 1A). There was no overlap between melanopsin^+^ and Brn3a^+^ RGCs (Fig. 3A,B) in either Bst^+/−^ or WT mice.

### Dysmorphic mitochondria and reduced oxygen metabolism in Bst^+/−^ mice

**Fig. 4.**
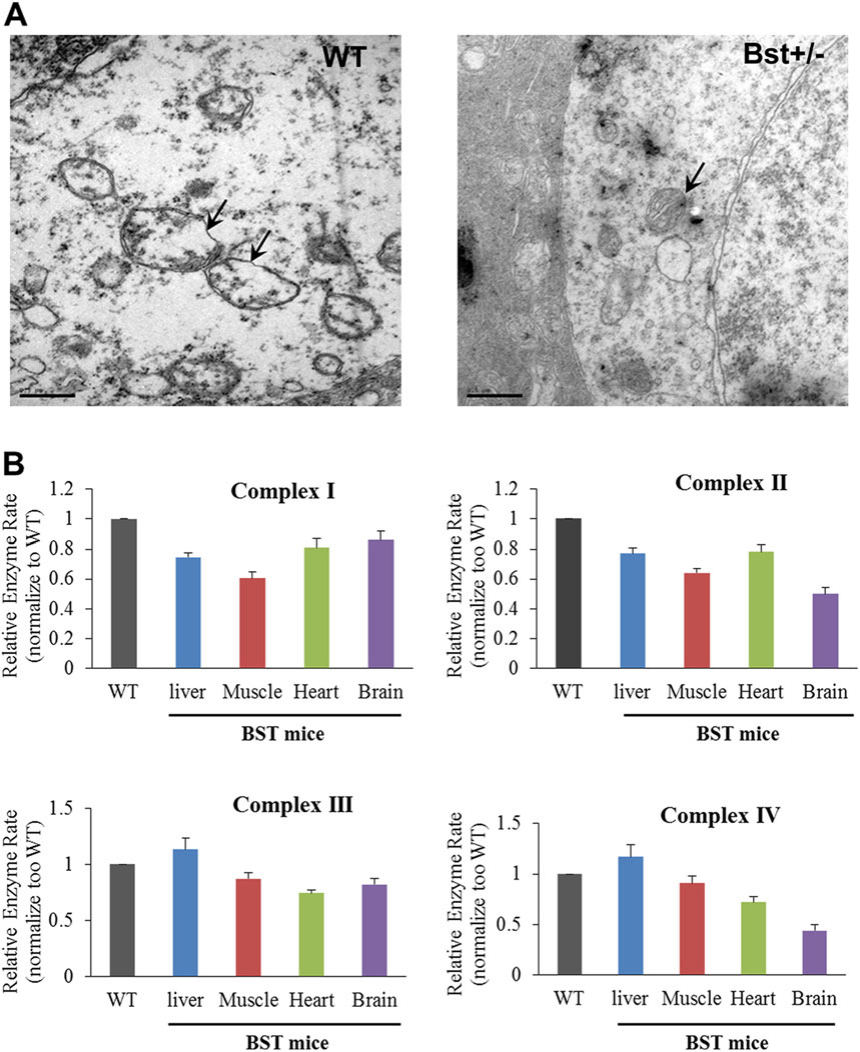
**Mitochondrial structure and function analysis.** (A) Mitochondria from retinal ganglion cells were observed to be deformed, with irregular borders lacking cristae in Bst^+/−^ mice, compared with WT mice. (B) Mitochondrial respiratory complex activities were measured on various tissue samples from WT and Bst^+/−^ mice. The activities of complex I-IV in most tissues were reduced in Bst^+/−^ mice, except for the liver. Error bars are s.e.m. Scale bars: 0.5 μm.

To examine the ultrastructure of the Bst^+/−^ retina, we performed EM on retina ganglion cells from Bst^+/−^. As shown in Fig. 4A, Bst^+/−^ mice had highly dysmorphic mitochondria with irregular borders lacking cristae compared with the ultrastructurally normal mitochondria in WT mice. This altered mitochondrial morphology in Bst^+/−^ mice was associated with a 60-80% reduction in oxygen consumption relative to that of WT mice (Table 1), with the deficit being variable across tissues. The morphological changes and reduced oxygen consumption is associated with abnormal electron transport complex activities. The mean activity of Complex IV in Bst^+/−^ heart tissue was reduced to 40% of the mean activity level observed with Complex IV proteins from WT mice (Fig. 4B). However, mitochondria complex activities in the Bst^+/−^ liver were similar to WT levels (Fig. 4B).

**Table 1.**
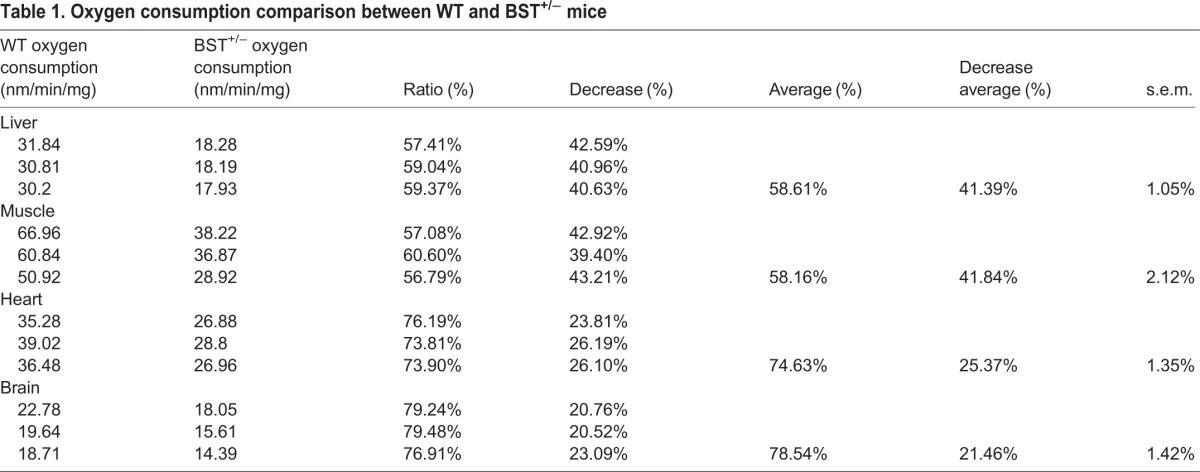
**Oxygen consumption comparison between WT and BST^+/−^ mice**

### Retinal vasculature development is delayed in the Bst^+/−^ mice

**Fig. 5.**
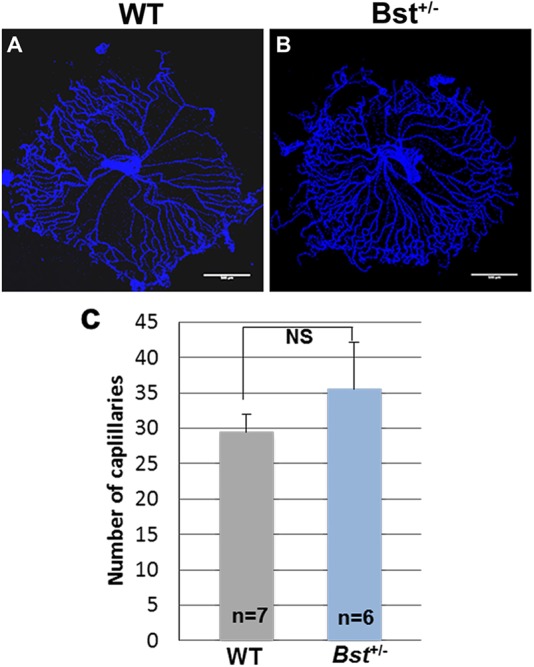
**Delayed hyaloid regression in Bst^+/−^ mice.** Hyaloid vessel in retinas collected from WT (A) and Bst^+/−^ mice (B) at postnatal day 8. (C) Quantitative analysis of capillaries. A circle was drawn around the hyaloid prep and the number of vessels crossing a concentric circle with half the radius was counted. Error bars are s.e.m. NS, not significant.

**Fig. 6.**
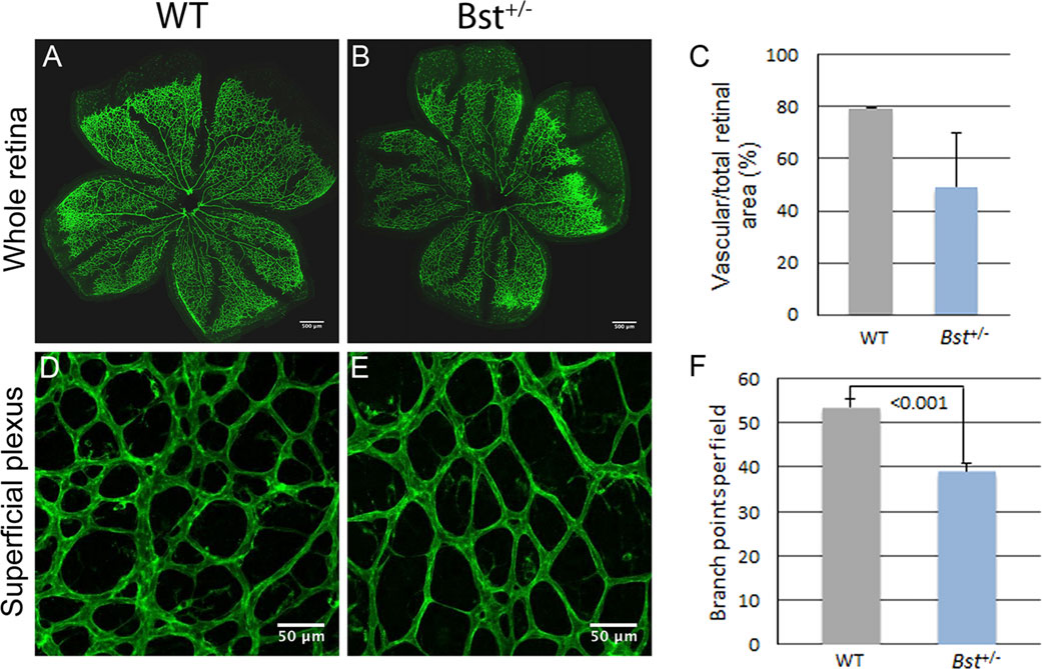
**Retinal angiogenesis.** Low- (100×; A,B) and high- (200×; D,E) magnification views of isolectin-labeled retinas from WT and Bst^+/−^ mice at postnatal day 8. Quantitative analysis of vascularized areas in whole retina (C) and branch points in superficial plexus (F). Error bars show s.e.m. Sample size *n* as indicated. *P*-values obtained by Student's *t*-test.

Examination of hyaloid vessels in mice sacrificed on postnatal day 8 demonstrated delayed hyaloid regression in Bst^+/−^ pups (*n*=6; Fig. 5A) compared with WT pups (*n*=7; Fig. 5B). Additionally, examination of isolectin-labeled vasculature in retina whole-mount specimens revealed that the vascular area of Bst^+/−^ retinas was reduced to ∼50% of the retina compared with ∼80% in WT retinas (Fig. 6C). WT retinas had 54 vessel branch points per microscope field on average, whereas Bst^+/−^ retinas had only 39 branch points per field (*P*<0.001) (Fig. 6F).

## DISCUSSION

The present study demonstrated that the Bst mutation affecting *Rpl24* alters cellular development and vascular patterning of the mouse retina. Although the Bst^+/−^ mice had normal appearing ipRGCs, they had severely reduced numbers of (presumably melanopsin^–^) RGCs overall, with abnormally thin IPL and INL. The presently observed absence of co-localization between melanopsin and Brn3a immunoreactivity, in both mutant and WT specimens, indicates that melanopsin^+^ and Brn3a^+^ RGCs are different populations of cells. Fewer than normal (i.e. than WT) retinal vessel branch points per viewing field were observed in Bst^+/−^ mouse pup retinas, indicating that they had a retinal angiogenesis defect. Bst^+/−^ mice exhibit abnormal PLR, but have preserved circadian rhythms. Finally, Bst^+/−^ mice were observed to have abnormal mitochondrial morphology, markedly reduced oxygen consumption and reductions in mitochondrial complex activities, indicating that mutation of *Rpl24* can result in dysmorphic and dysfunctional mitochondria.

Rods and cones were long considered to be the only mammalian photoreceptors until ipRGCs were discovered (Berson et al., 2002). ipRGCs appear to represent a small subset (∼1-3%) of the retinal ganglion cells (Fu et al., 2005). They have monosynaptic projections to the suprachiasmatic nucleus (SCN) and the intergeniculate leaflet (Pickard, 1985), responsible for circadian photoentrainment, and to the olivary pretectal nucleus, responsible for the PLR (Hattar et al., 2006). In the mouse, the rod-cone and melanopsin systems together seem to provide all photic input necessary for non-image-forming visual processes, including the PLR and circadian photoentrainment (Hattar et al., 2003). In addition to being directly photosensitive themselves, ipRGCs also receive synaptic input from rod-cone networks. They are the principal conduits for rod-cone input into non-image-forming vision.

The circadian rhythm is a cycle of physiological processes regulated by the SCN of the anterior hypothalamus through control of melatonin release from the pineal gland. ipRGCs provide primary light input to the SCN for regulation of the circadian rhythm (Ruby et al., 2002). Mice with degenerated rod and cone cells still exhibit normal circadian rhythms (Foster et al., 1991), but genetic ablation of ipRGCs can eliminate circadian photoentrainment (Güler et al., 2008). The normal circadian rhythms observed in Bst^+/−^ mice indicate that the ipRGC system involved in circadian rhythm regulation is functional in the mutant mice. Our finding of similar numbers of melanopsin^+^ RGCs in Bst^+/−^ and WT mice is consistent with the notion that ipRGCs are important for circadian rhythm timing.

On the other hand, the PLR was severely disrupted in the Bst^+/−^ mice. Photoreceptor signals from rods and cones, as well as directly from ipRGCs, are transferred to the visual cortex principally via ipRGCs. ipRGCs also project to the olivary pretectal nucleus, contributing to both the sympathetic and parasympathetic pupillary reflex pathways. The parasympathetic pupillary pathway relays via the Edinger–Westphal nucleus and the ciliary ganglion before reaching the iris muscles (Markwell et al., 2010). The intact circadian rhythm and normal-appearing melanopsin^+^ ipRGCs in the Bst^+/−^ mice suggests that the PLR dysfunction seen in these mice is due to developmental aberrations beyond melanopsin^+^ ipRGCs. Yet, it is possible that there is an ipRGC subtype specifically responsible for the PLR that is impaired in Bst^+/−^ mice. It has been reported that ablation of Brn3b^+^ ipRGCs disrupts the PLR severely without impairing circadian photoentrainment (Chen et al., 2011). Although any defects in PLR pathways could potentially affect the PLR, failure to differentiate a particular subtype of ipRGC remains a plausible mechanism underlying the ocular functional abnormalities observed in the Bst^+/−^ mouse.

Melanopsin^+^ RGCs are important for hyaloid vessel regression and angiogenesis during retina development (Ito and Yoshioka, 1999). In normal development, hyaloid regression clears the optical path during retinal angiogenesis, which is crucial for supporting the high metabolic demand of retinal neurons and is regulated by a melanopsin-dependent light response via VEGF pathway signaling (Rao et al., 2013). Although we observed apparently normal melanopsin^+^ RGCs in the Bst^+/−^ mouse retina, the Bst^+/−^ mouse retina exhibited delayed hyaloid regression and defective retinal angiogenesis, suggesting that Bst^+/−^ melanopsin^+^ RGCs have unknown functions during development.

Ocular neovascularization is the underlying cause of a number of retinal diseases, including proliferative diabetic retinopathy, retinopathy of prematurity and neovascular age-related macular degeneration (Klein et al., 1994). Currently, most animal models for subretinal neovascularization involve chemical or physical stimulation of subretinal vessel growth (Kimura et al., 1999; Okamoto et al., 1997), but available genetic models are insufficient to model RGC-loss diseases and with defects not suitable for further investigating various treatment schemes, such as stem cell therapy. For example, studies in Leber's hereditory optic neuropathy (LHON) mouse models have shown RGC axonal swelling, preferential loss of the smallest fibers, abnormal mitochondrial morphology, proliferation in RGC axons and decreased liver mitochondrial complex I activity (Lin et al., 2012). LHON mice have significant deficits in nearly all electroretinography (ERG) parameters, but do not exhibit reduced visual responses, and thus lack a simple readout for visual function that could be used in studies of the efficacy of potential therapies, including stem cell therapies. Alternatively, the heterozygous *Opa1* knockout mouse does show a disease phenotype, but not until the animals reach 18-20 months of age, and the homozygous *Opa1^−/−^* knockout is embryonically lethal (Davies et al., 2007).

Therefore, the present characterization of vascularization in the Bst^+/−^ mouse lays the foundation and provides important parameters for development of the Bst^+/−^ mouse into an ocular neovascularization genetic model. It would also be of interest to explore how stem cell transplantation might affect hyaloid regression and vascular development in Bst^+/−^ mice. Stem cell transplantation holds great potential for restoration of loss of RGCs due to retinal degenerative disorders, for which there is currently no effective treatment. The PLR pathway defect in Bst^+/−^ mice could serve as a simple read-out for the efficacy of stem cell transplantation. Thus, the Bst^+/−^ mouse might be a suitable preclinical model for retinal degeneration.

In conclusion, the present study demonstrates that the Bst^+/−^ mouse retina is characterized by a severe reduction in RGC quantity and a marked reduction in angiogenesis, with melanopsin^+^ ipRGCs remaining apparently intact and Brn3a- and melanopsin^+^ RGCs missing entirely. Additionally, Bst^+/−^ mice were found to have dysmorphic and dysfunctional mitochondria, which might provide an additional therapeutic target. Finally, Bst^+/−^ mice show normal circadian rhythms, but exhibit abnormal PLRs. Rescue of PLR in the Bst^+/−^ mouse could be used to demonstrate efficacy of stem cell transplantation.

## MATERIALS AND METHODS

### Animals

Bst*^+^*^/−^ and Bst^+/+^ C57/BlkS mice were obtained from Jackson Laboratory. All animal experimentation was carried out using protocols approved by the Institutional Animal Care and Use Committees at Cincinnati Children's Hospital Medical Center and University of California, Irvine, USA.

### Histology and IHC

Eye cups were dissected and immersed in 4% paraformaldehyde in phosphate-buffered saline (PBS) for 24 h and washed three times with PBS. Dissected eye cups were infiltrated with 30% sucrose overnight, embedded in OCT compound (International Medical Equipment) and frozen in isopentane on dry ice. Transverse sections of the retina (10 µm thick) were cut on a cryostat and mounted onto slides. Pre-IHC antigen retrieval was performed by incubating dried frozen sections for 20 min at 70°C with HistoVTOne (Nacalai USA). The frozen sections were then washed with PBS and blocked for at least 1 h in 20% goat serum in PBS. Primary antibodies used were anti-Brn3a (1:50; Santa Cruz Biotechnology, catalog number sc-8429) and anti-melanopsin (1:5000; Advanced Targeting Systems, catalog number AB-N38). Sections were incubated in primary antibodies overnight at 4°C and followed by three times washing with PBS. Primary antibody binding was detected by incubation with the following secondary antibodies, as appropriate, diluted 1:400 in PBS: Alexa Fluor 488 goat anti-rabbit IgG, Alexa Fluor 594 goat anti-rabbit IgG, Alexa Fluor 488 goat anti-mouse IgG and Alexa Fluor 594 goat anti-mouse IgG. The sections were counterstained with DAPI (40, 60-diamidino-2-phenylinode hydrochloride) and coverslipped with Vectashield (Vector Labs). Sections were imaged with a Nikon Ti Microscope (Nikon).

### Whole-mount retina immuno-fluorescence staining

On day 1 of a 7-day protocol, the eyes were removed and placed in 4% PFA for 45 min. Immediately after fixation, retinas were dissected and flattened by applying curve-relieving cuts. The retinas were then fixed for an additional 1-2 h. The retinas were removed from their slides and placed in 4% PFA in 1.6-ml tubes overnight at 4°C. On day 2, the retinas were washed twice with PBS and then blocked for 3 h in blocking solution (5% goat serum+0.3% Triton X-100 in PBS) at room temperature. The fixed, flattened retina specimens were incubated with anti-melanopsin antibody (same as that used for IHC, 1:5000 in blocking solution) for 4 days at 4°C. On day 6, the specimens were washed three times in PBS for 10 min and then left in PBS overnight at 4°C. On day 7, the specimens were incubated with goat anti-rabbit secondary antibody (1:1000 in blocking solution) for 3 h at room temperature, washed three times in PBS for 20 min and mounted on slides.

### Pupillary light reflex

All animals were kept on a 12 h:12 h light/dark cycle (12:12 LD) for at least 7 days before PLR testing. Before each experiment, the animals were dark-adapted for at least 1 h. While one eye received light stimulation with a specific intensity from a 470-nm light-emitting-diode light source (Super Bright LEDs), a digital camcorder (DCRHC96; Sony) recorded the other eye for 30 s (30 frames/s) under a 940-nm light. The percentage of pupil constriction was calculated as the percentage of pupil area at 30 s after initiation of the stimulus (steady state) relative to the dilated pupil size (right before light stimulation). The control and experimental animals are littermates.

### Circadian rhythm wheel-running activity

The same animals used in the PLR experiment were used in the wheel-running activity experiment. Mice were placed in cages with a 4.5-inch running wheel, and their activity was monitored with VitalView software (MiniMitter). The period was calculated with ClockLab (Actimetrics). Mice were kept on a 12:12 LD schedule for 2 weeks and then exposed to two ‘jet lag’ light manipulations: 10 days of a 6-h advance followed by 10 days of a 6-h delay. After the ‘jet lag’ paradigms, the mice were kept in constant darkness for 2 weeks followed by 10 days of constant light. Phase-shifting experiments were carried out on day 7 of constant darkness, in which each animal was exposed to a 15-min light pulse at CT16 (1500 lx). Animals were re-entrained to 12:12 LD for 2 weeks before being exposed to ultradian 3.5-h:3.5-h light/dark cycles. The intensity of light for all light phases was ∼1000 lx. Another set of mice was tested using a skeleton photoperiod, while two 1-h light pulses (800 lx), separated by 10 h of darkness, were administered.

### Isolation of mitochondria and functional analyses

Tissue mitochondria were isolated as described elsewhere (Hornig-Do et al., 2009). Briefly, tissue samples were minced into 1-2 mm^3^ pieces in ice-cold isolation buffer (10 mM EDTA/0.05% trypsin in PBS), incubated for 30 min on ice and centrifuged at 300 ***g*** for 5 min. The harvested pellets were homogenized in ice-cold PBS containing 10 mM EDTA and protease inhibitor with a GentleMACS Dissociator. The homogenized samples were filtered through 70-µm filters and centrifuged (4°C, 5 min at 800 ***g***). The resultant supernatants were diluted in ice-cold separation buffer, mixed with anti-TOM22 MicroBeads and enriched on a MACS column. The magnetically labeled mitochondria were eluted for assays.

### Oxygen consumption assay

Oxygen consumption rate was determined as described previously (Stanley, 1986). Tissue mitochondria were diluted in 1 ml of warm (37°C) Medium A (full deficient medium with 0.01% CaCl_2_, 0.0105% isoleucine, 0.003% methionine, 0.0124% NaH_2_PO_4_·H_2_O, 2% dialyzed FBS and 0.01% pyruvate). The samples were loaded into a closed chamber in a Hansatech Oxygraph (Hansatech Instruments), and oxygen consumption was recorded. The instrument was calibrated with oxygen-saturated distilled water (217 nmol/ml) at 37°C.

### Measurement of respiratory chain complexes I-IV activity

The activities of the individual respiratory chain complexes I-IV were measured in isolated mitochondria as described previously (Hornig-Do et al., 2009). Complex I activity was assessed by following the decrease of NADH absorbance at 340 nm, with decylubiquinone as an electron acceptor. Complex II activity was measured by following the reduction of 2,6-dichlorophenolindophenol (DCPIP), as evidenced by decreases in the absorbance at 600 nm of the oxidized DCPIP. The activities of complex III and IV were determined by measuring the reduction and oxidation of cytochrome c, as reflected by increased and decreased absorbance at 550 nm, respectively. The activity data were normalized relative to citrate synthase activity.

### Hyaloid and retinal labeling and quantification

Hyaloid vessels were collected and stained with DAPI as described elsewhere (Lobov et al., 2005). Retinal flat-mounts were prepared and labeled with isolectin (Gerhardt et al., 2003). Hyaloid vessel quantification was conducted as described previously (Ito and Yoshioka, 1999). Retinal vessel density was quantified by counting vessel junctions with ImageJ in microscope fields at 200× magnification. Depth-coded three-dimensional image reconstructions were generated with a Zeiss Apotome-equipped microscope in conjunction with Axiovision software. Anti-calretinin antibodies (Millipore) were used for labeling of retinal flat-mounts.

### Data analysis

Quantitative data were compared between the Bst*^+^*^/−^ and WT groups with Student's *t*-test. Mean values are reported with standard errors of the mean (s.e.m.) error bars in the Figures.
